# Fully Implantable Low-Power High Frequency Range Optoelectronic Devices for Dual-Channel Modulation in the Brain

**DOI:** 10.3390/s20133639

**Published:** 2020-06-29

**Authors:** Woo Seok Kim, Minju Jeong, Sungcheol Hong, Byungkook Lim, Sung Il Park

**Affiliations:** 1 Department of Electrical and Computer Engineering, Texas A & M University, College Station, TX 77843, USA; wooseok.kim@tamu.edu (W.S.K.); hyhaerong@tamu.edu (S.H.); 2 Neurobiology Section, Division of Biological Sciences, University of California, San Diego, CA 92093, USA; jminju@ucsd.edu (M.J.); bklim@ucsd.edu (B.L.); 3 Department of Biological Sciences, Korea Advanced Institute of Science and Technology, Daejeon 34141, Korea; 4 Institute for Neuroscience, Texas A&M University, College Station, TX 77843, USA; 5 Center for Remote Health Sciences and Technologies, Texas A&M University, College Station, TX 77843, USA

**Keywords:** wireless optoelectronics, brain insertion device, magnetic field enabled devices, toggle logic circuit, flexible electronics

## Abstract

Wireless optoelectronic devices can deliver light to targeted regions in the brain and modulate discrete circuits in an animal that is awake. Here, we propose a miniaturized fully implantable low-power optoelectronic device that allows for advanced operational modes and the stimulation/inhibition of deep brain circuits in a freely-behaving animal. The combination of low power control logic circuits, including a reed switch and dual-coil wireless power transfer platform, provides powerful capabilities for the dissection of discrete brain circuits in wide spatial coverage for mouse activity. The actuating mechanism enabled by a reed switch results in a simplified, low-power wireless operation and systematic experimental studies that are required for a range of logical operating conditions. In this study, we suggest two different actuating mechanisms by (1) a magnet or (2) a radio-frequency signal that consumes only under 300 µA for switching or channel selection, which is a several ten-folds reduction in power consumption when compared with any other existing systems such as embedded microcontrollers, near field communication, and Bluetooth. With the efficient dual-coil transmission antenna, the proposed platform leads to more advantageous power budgets that offer improved volumetric and angular coverage in a cage while minimizing the secondary effects associated with a corresponding increase in transmitted power.

## 1. Introduction

Optogenetics is one of the most powerful genetic tools and enables the interrogation of neural function by targeted gene expression and activation of light-sensitive proteins [1,2,3]. Many neuroscientists are using this emerging generic tool to better understand how groups of interconnected neurons work together to carry out behaviors [4,5,6]. In a typical experiment setup, a mouse is tethered to a remotely located light source such as a laser or light-emitting diodes (LED), and light is delivered to targeted brain regions through an optical fiber [2,3]. Unfortunately, such an optical fiber-based approach imposes physiological or psychological stresses on animals and thereby makes it less ideal for longitudinal experiments [7]. Wireless optogenetic systems can enable experiments in ways that are difficult with optical fibers [6,8]. Most existing wireless technologies allow for a single-channel operation in high frequency (HF)/ultra-high frequency ranges (UHF) or dual-channel operation in UHF ranges [9,10,11,12,13]. Microcontroller (µC) embedded communication systems, near field communication (NFC), or Bluetooth chips could achieve multichannel operation [14,15,16]. Although such wireless platform systems provide some utility with user-friendly software, the power requirements for wireless operation (~3 mA for a µC embedded communication system, ~3.5 mA for an NFC chip, and ~10 mA for a Bluetooth device) make them less ideal for small animal research or longitudinal experiments [17,18,19,20]. Furthermore, the wireless power transmitter for NFC or Bluetooth systems must deliver a power level of 8–12 W in order to activate the devices in a cage, and this could exceed the upper limits suggested by IEEE depending on the classes of neuroscience experiments [21]. Any exposures above the guidelines could potentially cause tissue damage associated with the absorption of radio-frequency (RF) signals [22]. 

In comparison with the multichannel operation enabled by µC, NFC, or Bluetooth chips, a customized toggle logic circuit that permits switching or channel selection requires <300 µA for its wireless operation [23]. Hinged on this approach, we integrated low-power control logic circuits with a reed switch into a fully implantable optoelectronic device. This soft, miniaturized low power optoelectronic device yields versatile capabilities and a simultaneous/independent control over multiple light sources with emission wavelengths across the visible range in an individual animal. Here, we suggest two different dual-channel devices based on the mechanism of actuating the reed switch in different ways, utilizing the nature of the reed switch reacting to the electromagnetic field. In addition, by efficiently applying the dual-coil transmission (TX) antenna, it is possible to supply a sufficiently powerful and stable wireless power transmission even at a lower RF power level (a transmitted power level of 1–2 W). Combining the proposed TX system and low power consumption logic devices, we demonstrate the utility of the wireless dual-channel devices via in vitro and in vivo experiments, which expand the potential functionality and advanced operational modes for their widespread use in a variety of neuroscience research. Systematic electromagnetic simulation studies establish effective TX antenna designs and a range of operating conditions for animal studies. The sham study using controlled groups of mice reveals that the implantation of the proposed system does not alter the animal’s behavior.

## 2. Materials and Methods

### 2.1. Device Fabrication

**Figure 1:**
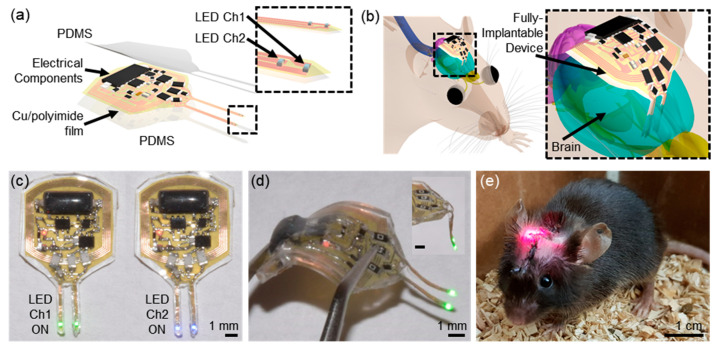
System overview. (**a**) Schematic illustration of a soft, fully implantable dual-channel optogenetic device. (**b**) Illustration of the device location relative to the brain. (**c**) Demonstration of a dual-channel wireless operation. (**d**) Picture of the device after bending the body of the device. (**e**) Image of a mouse with the device implanted.

We start with a flexible copper (Cu, 18 μm thickness) and polyimide (PI, 12 μm thickness) substrate on the glass (dimensions, 5.08 cm by 7.62 cm), and deposit photoresist (spin-coating at 3000 rpm for 30 s and baking on a hot plate at 100 °C for 4 min) on the film in the clean room. We use the UV photo-lithography (UV intensity for 265 mJ/cm^2^) tool to define patterns, and these patterns are immersed in the developer solution for 30 s and in copper etchant for 5 min. Rinsing the patterns with acetone, isopropanol (IPA), and distilled (DI) water for 10 s, respectively, we then obtain the base patterns of the device. After the patterns are dried, placed and soldering miniaturized, the electronic components are surface-mounted, including the passive circuit components, micro-scale LEDs (µ-LEDs), a reed switch, an analog switch, and transistors, on a flexible substrate, yielding the electronic circuit on the patterns. Next, the stainless-steel wire (6 mm length) that provides stiffness during insertion is attached to the backside of the deep brain insertion probe by a biocompatible UV epoxy. This guide steel applies only to the µ-LED probe, and this remains inside the brain when implanted. For the last step, the whole device is encapsulated with polydimethylsiloxane (PDMS; 10:1 mix ratio, 50–100 µm thickness), followed by baking in an oven at 100 °C for an hour; detailed procedures of fabrication and equipment information are found in Table 1. The final dimension of a device is 10 (w) × 10 (l) × 1 (h) mm, and the diameter of probes is <400 µm (Figure 1a,b). The weight of an entire device (~250 mg) is approximately a hundred and twenty times lighter than the weight of a mouse (∼30 g). These processes lead to a flexible, transparent, and biocompatible platform for the wireless delivery of light to deep brain circuits (Figure 1c,d). A red LED serves as an indicator to ensure the device operation when implanted in an animal (Figure 1e). Detailed information on the device layouts and electrical components are shown in Figures S1 and S2.

### 2.2. Actuation Mechanisms by a Magnet

A circuit diagram is shown in Figure S3a. Assuming that Channel 1 is on and Channel 2 is off (Figure S3b), a reed switch responds to a magnet and makes physical contact when it is close to the reed switch. Once the reed switch, S1, is close, electrical charges stored at C1 discharge through Rx (Figure S3c). This brings a gate voltage at a node of V_N2-G_ to the threshold voltage for the NMOS2 transistor (Figure S3d). It immediately turns on the NMOS2 transistor (Figure S3e), and a drain voltage of the NMOS2 transistor at a node of V_Sel_ drops down to the ground level (Figure S3f). Since the PMOS transistor shares its node for the gate with the NMOS2 transistor, the PMOS transistor turns on (Figure S3g), and its drain node, V_P-D_, becomes high enough to turn on the NMOS1 transistor (Figure S3h). This ensures the activation of the NMOS1 transistor and prevents charge buildups at C1 (Figure S3i). This leads to the activation of Channel 2 (Figure S3j). The reed switch allows for a reversible activation/deactivation wireless operation. Since no charges are accumulated at C1, its voltage level is zero (Figure S3j). When we place a magnet to the device in an attempt to select Channel 1, the reed switch is activated again (Figure S3k). This causes a gate voltage at a node of V_N2-G_ to move away from the threshold voltage for the NMOS2 transistor (Figure S3l). This shuts off the NMOS2 transistor (Figure S3m), and the drain voltage level at a node of V_Sel_ becomes high enough to turn off the PMOS and NMOS1 transistor (Figure S3n). Consequently, the high level of voltage at V_Sel_ activates Channel 1, and the LEDs for Channel 1 light up (Figure S3o).

### 2.3. Actuation Mechanisms by a Radio-Frequency Signal

A circuit diagram appears in Figure S4a. When the TX system transmits continuous power signals, the voltage level (or gate voltage of the NMOS1 transistor) at a node of V_1_ is high enough to turn on the NMOS1 transistor (Figure S4b). This is because no current path exists between R1 and a reed switch. Since the PMOS and NMOS2 transistors share their nodes, gate, and drain, respectively, with the NMOS1 transistor, a high level of voltage at V_1_ ensures the deactivation of the PMOS and NMOS2 transistors (Figure S4c). Consequently, a voltage level at V_1_ stays constant and high during the operation by electrical charges stored at C1 (Figure S4c). This results in the activation of Channel 1 for inhibition and the deactivation of Channel 2 for stimulation (Figure S4d). For the stimulation of neuronal populations, pulse signals must be delivered [24], and the following explains the procedures for stimulation. When the TX system sends a train of pulse signals, alternating electromagnetic (EM) fields or rectified AC signals trigger the reed switch (Figure S4e). Once the reed switch is triggered, V_1_ is grounded (Figure S4f), and subsequently NMOS1 shuts off. This results in the deactivation of Channel 1 for inhibition (Figure S4g) and simultaneously turns on the PMOS transistor (Figure S4h). Consequently, the activation of the PMOS transistor brings the voltage level at a node of V_2_ to the threshold voltage for the NMOS2 transistor, and the NMOS2 holds its drain voltage to the ground or zero during the operation (Figure S4i). This ensures the activation of Channel 2 for stimulation (Figure S4j).

### 2.4. Finite Element Analysis

For the numerical electromagnetic simulations of the proposed device and TX system, we used a finite element analysis tool, Ansys Electromagnetics Suite 17-HFSS, to look for the strength and uniformity of the electromagnetic field in the mouse’s homecage (dimensions, 16 (w) × 25 (l) × 13 (h) cm). Organ systems and tissues of a mouse were modeled to one million meshes for numerical simulations, and antenna coils made of copper stripes were modeled to materials with a finite conductivity, 58 MS/s. Most existing wireless powered optoelectronic devices have power delivery issues of angle and position dependency in a large area that allows experiments with a freely moving animal. To prove that we overcame this matter in our proposed system, we elicited simulation results in various positions and angles of the proposed device in the homecage with the dual-coil TX system. All simulations were conducted with the 2 W, TX power level as a result of considering the safety level of electromagnetic fields [21].

### 2.5. The Coverage Measurements of the Wireless Power TX System

We implanted a wireless device with a red-colored LED over the skull, under the skin of a mouse, and recorded its behaviors using two webcams (C615, Logitech, Lausanne, Switzerland). This red LED mounted device serves as an indicator that can be detected by webcams over a cage when the wireless TX system transmits RF signals at a level of 1 W. Two webcams at different angles (top and sideward) recorded the behaviors of an animal in a cage for two minutes with 24 frames/s at HD resolution. By extracting images from the recordings and analyzing the image frame by frame, we counted whether the image captured the red LED flash from the implanted device in the mouse’s head. That is, we calculated the ratio of frames that were missing or captured the wireless device’s operation over the entire frames.

### 2.6. Procedures for Device Implantation and Sham Study

All procedures to maintain and use mice were approved by the Institutional Animal Care and Use Committee (IACUC) at the University of California, San Diego. 8–12 weeks old C57BL/6 mice were used. The mice were maintained on a 12-h:12-h light:dark cycle with ad libitum access to food and water. Mice were anesthetized with a mixture of ketamine (100 mg/kg) and dexmedetomidine (1 mg/kg) and placed in a stereotaxic apparatus (David Kopf Instruments). Holes at coordinates corresponding to the ventromedial hypothalamic nucleus, ventrolateral part (VMHvl) (anteroposterior, −1.5 mm; mediolateral, ±0.6 mm; dorsoventral, 5.0 mm from top of the skull) were made by a dental drill. For the μ-LED probe implantation group, μ-LED probes were bilaterally inserted into the brain and fixed to the skull using adhesive cement (C&B metabond, Parkell, Edgewood, NY, USA). Then, the body of the device was located onto the skull and secured using dental cement. For the control groups, only the device without the μ-LED probes was implanted onto the skull, and wild-type mice with no implantation were also tested. After 3–5 days of recovery from surgery, each mouse was placed at the center of a square acrylic box (27.5 (w) × 27.5 (l) × 30.5 (h) cm), and freely explored for 40 min. Locomotor activities were recorded with a Logitech C920 camcorder in a dark room. The recorded videos were analyzed using the EthoVision XT 13 software (Noldus Information Technology, Wageningen, The Netherlands). For the histological analysis, mice were anesthetized with isoflurane and perfused with 0.9% saline first and then with 4% paraformaldehyde (PFA) diluted in phosphate-buffered saline (PBS). The brains were removed and post-fixed overnight at 4 °C. 60 µm thick coronal sections were sliced on a vibratome (VT100S, Leica, Wetzlar, Germany) and mounted onto glass slides with DAPI mounting solutions. All images were acquired with an Olympus FluoView FV1200 confocal microscope. 

### 2.7. Measurements of Durability and Heat Dissipation

We performed device lifetime testing where a device is immersed in 10% PBS solution at various temperatures (36 °C, 60 °C, and 90 °C), and the light intensity was measured daily using a light meter (LT300, Extech) (Figure S5a). Additionally, we monitored a red LED embedded in a wireless device that serves as an indicator of the wireless operation when implanted (Figure S5b). For the heat dissipation of the proposed devices, we used an infrared camera (VarioCAM HDx head 600, InfraTech, Dresden, Germany), and the device was in a wet condition by a 10% PBS solution. The light intensity of the μ-LED was fixed at 10 mW/mm^2^, which is enough to activate light-sensitive proteins, and the camera recorded temperature changes when the device was served as a 200-ms pulse with various duty cycles: 0% (off), 20%, 40%, 60%, 80%, and 100% (always on).

## 3. Results

In this study, we introduce a soft, fully implantable miniaturized low-power optoelectronic device that operates in HF ranges with powerful capabilities to simultaneously/independently modulate discrete circuits in the brain of a freely-behaving animal through a dual-coil TX system which consists of a customized dual-coil antenna and commercial 13.56 MHz RF signal generators (LRM-2500A, FEIG electronic, Weilburg, Germany). This proposed TX antenna provides a more efficient transmitted power than existing antennas at the same TX power level. With this system, the optoelectronic device that is supplied with power by the RF signals only is able to secure a more stable power supply. In addition, since our fully implantable device is an embedded logic circuit including a reed switch, not integrated circuit (IC) packages, it allows for a straightforward wirelessly dual-channel modulation in a manner that provides more beneficial power budgets as well as keeping biological tissues together. A thermal assessment of wireless devices using an infrared camera revealed that no detectable change (<0.3 °C) in temperatures during operation was observed in Figure S5c, suggesting that heat generation from LEDs is not detrimental to the operation of LEDs. Additionally, the computed SAR distribution on a mouse model showed that <12 mW/kg is below the guidelines suggested by IEEE (Figure S5d) [21]. 

### 3.1. Low-Power, Magnet Induced Dual-Channel Optoelectronic Device

**Figure 2:**
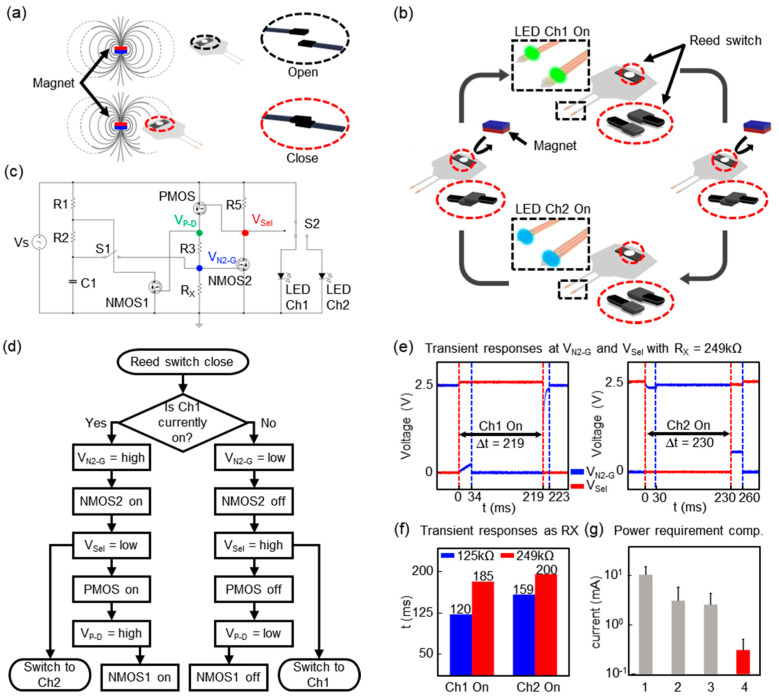
Electrical Characteristics of a dual channel wireless optogenetic device. (**a**) Essential feature of a reed switch. (**b**) Illustration of procedures for a channel selection of the proposed device by a magnet. (**c**) Circuit diagram of the control logic including a reed switch and LEDs. The reed switch and an analog switch IC are denoted by S1 and S2, respectively. V_P-D_, V_N2-G,_ and V_Sel_ represent a level of voltages at the PMOS drain, NMOS2 gate, and a selection input port of an analog switch, respectively. The rest of the parameters for capacitors and resistors are C1 = 1 µF, R1 = R2 = 249 kΩ, R3 = 10 kΩ, R5 = 20 kΩ. (**d**) A flowchart for an actuation mechanism enabled by a reed switch. (**e**) Plots of the transient responses at V_N2-G_, and V_Sel_, respectively, during the transition from Ch 1 to Ch 2 (Left) or vice versa (Right). Here, the dotted lines indicate the moment at which S1 closes (Red) or opens (Blue). (**f**) Plot of the measurements of the threshold time for switching as a function of R_x_. (**g**) Comparisons of the power consumption of the proposed system (denoted, 4) with that of µC (denoted, 3), NFC (denoted, 2), and Bluetooth (denoted, 1) devices, respectively.

Figure 2a highlights the essential electromagnetic properties of a reed switch. The reed switch is an electrical switch controlled by an applied magnetic field, and it consists of a pair of ferromagnetic flexible metal reeds contacts [25]. The contacts are normally open, and they are close when a magnetic field induced by a magnet or alternating electrical signals is present. Figure 2b illustrates the procedures for a channel selection by a magnet, and a circuit diagram is shown in Figure 2c. Here, the device includes an actuating mechanism, enabled by a magnet, configured to switch a channel to the other one, from Channel 1 to Channel 2 or vice versa in response to a magnet (Video S1). Previous research demonstrated the effective range in which the device’s reed switch was activated by various magnets [23]. In this study, a 0.45 T magnet was used, which could actuate the reed switch in the implanted device within ~5 cm. In Video S1, a 0.45 T magnet was attached to the outside of the cage wall near the water well; it shows an example of an animal experimental design, such as modulating dual-channel optical stimulation in the target region whenever the mouse drinks water. This results in a simplified dual-channel wireless operation. A flowchart for actuation mechanisms and circuit diagrams with signal-flows from a power source to LEDs that capture the transient responses during switching appear in Figure 2d. The device layout and information on all the electrical components are shown in Figure S1, and detailed actuating signal flows are found in Figure S3. The measurements of the transient responses reveal that the switching time required for channel activation is a function of the time constant, R_x_ × C1 (Figure 2e,f). This indicates that the switching time must be long enough so that the device is activated or deactivated, and that the device remains unchanged when the switching time is shorter than the threshold, defined by R_x_ × C1. Although a device may activate or deactivate in the presence of electromagnetic interferences or noises, the tunable nature ensures a robust device operation in a manner that extends the switching time long enough and therefore offers insensitivity to surroundings or environmental electromagnetic noises. Figure 2g highlights the essential feature of the device, a low-power wireless operation for channel activation/deactivation, and comparisons with the power requirements for a multichannel operation enabled by µC embedded communication, NFC, or Bluetooth systems [14,15,16]. The measurement result reveals that the proposed logic circuit requires <300 µA for switching or channel selection, while the datasheet or white papers of µCs (ATTINY84A, ATMEL^®^ & MKL03Z32VFG4, NXP Semiconductors), NFC (M24LR64E-R, ST microelectronics), or Bluetooth (nRF51824, Nordic semiconductor) devices indicate at least 2.5 mA for a multichannel operation [17,18,19,20]. A low-power wireless operation allows for enhanced wireless coverage more than any other existing wireless platform devices do at a given transmitted power level (below 2 W).

### 3.2. Magnet-Free, Dual-Channel Optoelectronic Device

**Figure 3:**
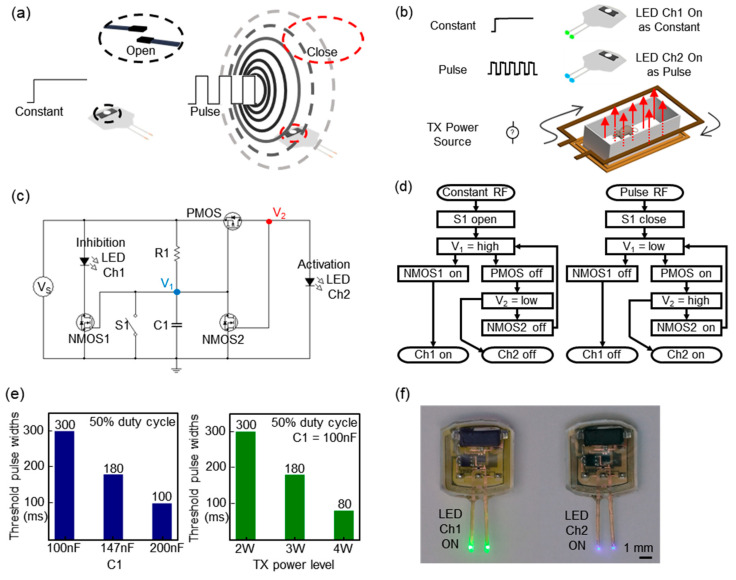
Demonstration of an advanced operational mode and magnet-free operation to expand the function and its characteristics. (**a**) Illustration of a mechanism by which a train of pulse signals induces an alternating magnetic field. (**b**) Schematic illustration of a magnet-free operational mode: inhibition and stimulation of neural activity. (**c**) Circuit diagram of a dual channel device that offers an advanced magnet-free operational mode: inhibition and stimulation of neural activity. Here, a dual channel device automatically activates a channel in response to signals from a remotely located wireless TX system. R1 = 20 kΩ and the reed switch is denoted by S1. (**d**) Flowchart that depicts an actuation mechanism enabled by a pulse signal. (**e**) Plot of the measurements of the threshold pulse widths required for the switching operation as a function of a capacitor, C1, and TX power. (**f**) Pictures of an advanced operational mode and magnet-free operation.

The platform can also offer a magnet-free operation mode and be extended to include a design that allows for the independent/simultaneous control of two channels: one channel is for stimulation, and the other is for inhibition. Here, magnetic fields that control a reed switch are generated by alternating electrical signals (AC or pulse signals) from the TX system (Figure 3a) [26]. The constant power signal generates static magnetic fields, which do not trigger a reed switch, while a pulse signal induces alternating magnetic fields sufficient to activate a reed switch. We utilize pulse signals as a source for the control of a reed switch; the antenna coil in the implantable device receives RF power from the TX system, rectifies voltages, and powers the remaining internal circuits of the device (Figure 3b). Details of its signal-flows and the circuit diagram appear in Figure 3c,d. Figure 3e shows the electrical characteristics of a switching circuit, including a reed switch. The capacitor, C1, and the levels of the transmitted power determine the threshold pulse widths for the activation of Channel 2 for stimulation, and this indicates that the TX system needs to send a longer pulse than the threshold for activation. Similarly, at high levels of TX power, the amplitudes of the rectified voltages in a wireless device are high enough to deliver the same amount of charges required for the activation of Channel 2 within a shorter pulse duration than the threshold at a low level of TX power and facilitate switching [27]. During inhibition or the continuous mode of operation, this could suggest possibilities of unstable operation or unwanted switching of the channel associated with environmental electromagnetic noises or interferences. However, its tunable nature can ensure a robust stability in operation with a strategy that allows a high threshold for activation. Once the RF power is shut off and restarted, Channel 1 becomes active again. During each operation, the non-targeted channel becomes disabled, and the LED in the non-targeted channel remains off (Figure 3f). Visual evidence confirms that cross-coupling between two channels is negligible, and the in vivo validation of the proposed magnet-free dual-channel operation in an open field experimental assay demonstrates the versatilities of the technology in new classes of neuroscience studies (Video S2).

### 3.3. Wireless Coverage

**Figure 4:**
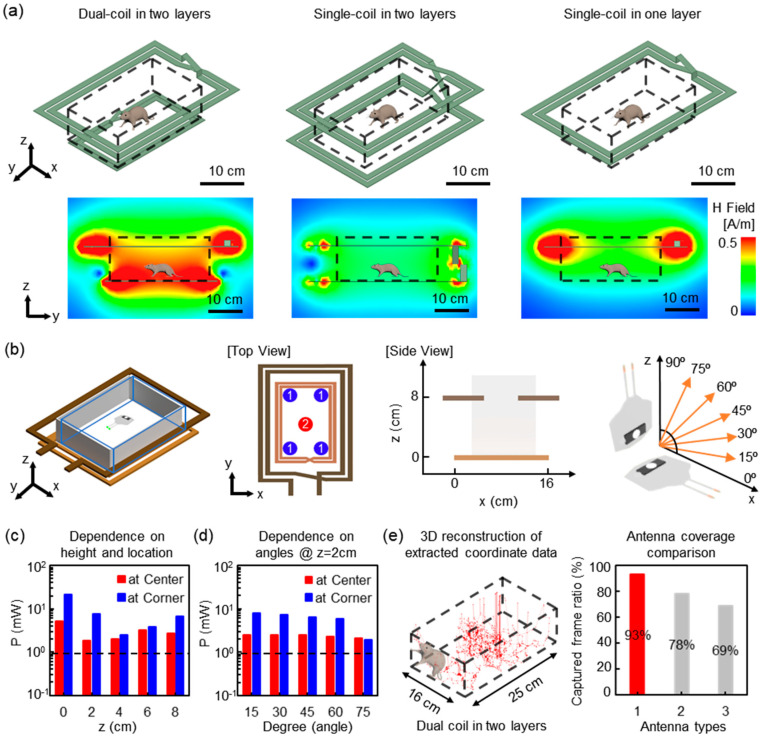
Modeling and experimental results for the power transmission from antennas and angular coverage. (**a**) Electromagnetic simulations of the wireless power transmission efficiency for three different TX systems. (**b**) Illustration of an experimental cage for evaluating an implantable device with a dual coil TX system. (**c**) Plots of the measured electrical power as a function of the position along the Z-axis at a corner and at the center of the cage, respectively. (**d**) Plots of a residual dependence of the transmitted power on the relative orientation angle between the transmission antenna and the implantable device. Dotted lines indicate the threshold electrical power (0.9 mW) required for the activation of light-sensitive opsins; 0.9 mW electrical power corresponds to an optical power of 15.7 mW/mm^2^. (**e**) 3D representative plot of post-processed reconstructed data from extracted coordinate information from two recorded videos (Left), and comparison of wireless coverage (Right) of dual-coil in two layers (denoted, 1) with that of single-coil in two layers (denoted, 2) and single-coil in one layer (denoted, 3).

The wireless power TX system consists of two coils, a source coil and a primary coil (Figure 4a on the left, and Figure 4b) [28,29]. The source coil that is connected to a signal generator (not shown in Figure 4) wraps around a cage, and the primary coil is placed underneath a cage. The primary coil is comprised of purely reactive components, a capacitor, and an inductor. The way RF signals are transmitted is that a signal generator delivers RF power at the frequency of 13.56 MHz to the source coil, and the RF power is transmitted to the primary coil. Finally, the implantable wireless device in a cage harvests power from the primary coil. The devices resonate when the resonant frequency is matched to that of the TX system. This results in more efficient energy harvesting [30,31,32]. Moreover, the dual coil TX system offers high quality (Q) factors compared with the modest Q factor of the single-coil TX system (Figure 4a) [29,33]. The simulation results reveal that the proposed dual coil TX system provides more uniform magnetic field distributions throughout the volume of a cage, while magnetic fields in the single-coil structures are only available near the coil. Systematic experimental studies and electromagnetic simulation using a commercial tool, HFSS, establish a range of effective operating conditions for animal behavior studies (Figure 4). Figure 4b illustrates an experimental cage for evaluating a dual coil TX system in terms of power transmission efficiency. The electrical power delivered to the implantable device varies with the angle of rotation of the devices around the y-axis, as illustrated in Figure 4b. Variations in power according to the position along the z-axis are acceptable for a range of distances that correspond to the size of a typical mouse (Figure 4c). Electromagnetic simulation represents a residual dependence of the transmitted power on the relative orientation angle between the transmission antenna and the implantable device (Figure 4d). In order to prove the advances of this dual-coil TX system, a wireless optoelectronic device is implanted on the skull of a mouse, and light emitted from the device through the skin is an obvious marker to determine wireless coverage. Figure 4e (Left) represents a 3D tracing image of reconstructed coordinates from the recorded data. Based on the computation of the captured frame ratio of the regular operating device in the entire frames, we come to the rational conclusion of the proposed type’s merit in terms of wireless coverage when compared to other designs (Figure 4e on the right). Collectively, the dual coil TX system enables the robust activation of a device in a cage.

### 3.4. Sham Study

**Figure 5:**
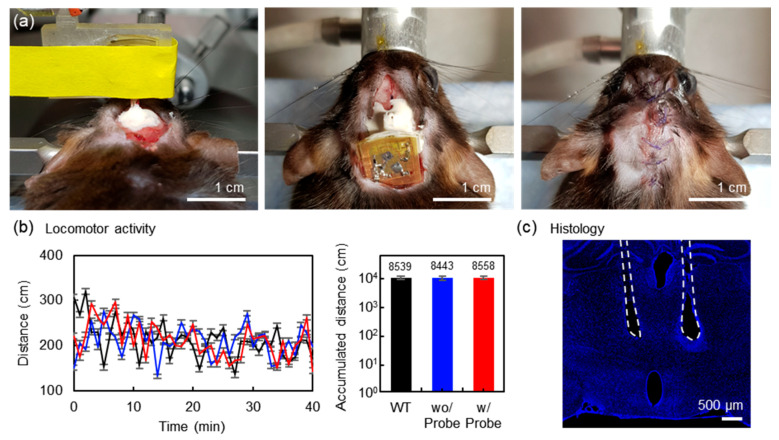
Surgical procedures for implanting the device for operation in the deep brain and locomotor activity. (**a**) Images of the surgical steps for holding, positioning, and injecting the body of the device. (**b**) Comparisons of the locomotor activity of the implanted device with probes (Red; n = 4), that of wild type mice (non-surgery; Black; n = 4), and that of the implanted device without probes (Blue; n = 4). The y-axis of the bar graph (Right) is a logarithmic scale. (**c**) Histology of a mouse brain with the device implanted. Dotted lines represent the placement of probes in the brain.

To determine whether the implantation of wireless optoelectronic devices affects animal behavior, we implanted the device with or without probes into wild type mice and performed an open field test (Figure 5a). Both the probe implantation and control groups (group 1; without probe implantation and group 2; wild type with no implantation) showed no difference in accumulated distance (Figure 5b), indicating that the μ-LED probes implantation does not affect locomotor activities. In addition, the histological results suggest that the μ-LED probes caused a similar level of mechanical damages to the conventional optic fiber implantation (Figure 5c) [34]. After establishing a mouse home cage and open field in the TX system, we also confirmed that light stimulation was successfully generated while the mouse was freely moving, and behavioral animal studies using the proposed system are ongoing (Video S3). Together, these results demonstrate that the proposed dual-channel implantable wireless optoelectronic device can be an essential tool for the optogenetic manipulation of neuronal activities with versatile animal behavior paradigms.

## 4. Discussion

A multichannel operation can be accomplished by commercially available µC embedded communication systems, NFC, or Bluetooth chips. The benefit of employing these chips in implantable applications is their user-friendly interface and software, and/or “easily understandable” characteristics of HF range electromagnetic waves, allowing researchers with little or no expertise in RF electronics to utilize the technologies for their experiments [15]. However, these solutions demand considerable power requirements for a wireless operation (>10 mA or 30 mW), and the wireless TX system must deliver a transmitted power level of 8–12 W to activate the device in a cage [16,20]. Specifically, the implementation of an antenna coil for µC, NFC, or Bluetooth chips at given dimensions (1 cm by 1 cm) or something comparable to the brain of a mouse could be challenging depending on the classes of neuroscience studies, such as longitudinal experiments that record neural activity over long period in a freely-behaving animal. Although large coil designs (>3 cm by 3 cm) and/or a high level of TX power (>10 W) with an advanced RF power delivery scheme can offer increased wireless coverage, improvement is marginal due to the nature of electromagnetic waves at HF ranges [16]. In other word, the generation of a magnetic field focuses on regions near a coil, and therefore harvesting efficiency at the center of a cage or TX coil is modest [35]. However, the proposed technology enabled by a reed switch and a control logic circuit allows for a low-power dual-channel operation (<300 µA for switching). The comparison table of the above studies is found in Table S1.

The straightforward extension of the platform is the optogenetic induction of bidirectional long-term synaptic plasticity at corticostriatal afferents within the dorsomedial striatum (DMS), a brain region involved in drug and alcohol addiction [36]. The ability for an independent control over two light sources such as a blue and red LED will enable experiments that demystify a causal link between synaptic plasticity and alcohol-seeking behavior via recently developed light-sensitive proteins, currently inaccessible due to limitations associated with existing hardware approaches [12,13,37]. In this setting, a magnet that generates a magnetic field for the control of a reed switch can be installed into an operant chamber equipped with photo interrupter sensors serving as nose pokes or an open field assay with a drinking well. The operant box or open field assay is widely used for the study, training animals by rewarding behavior related to various situations and external stimuli. When an animal with an implanted dual-channel device approaches ports or drinking wells in the assay, a magnet installed together with the port or well can remotely trigger the reed switch embedded in the wireless device, and discrete neuronal populations of interest can be selectively photostimulated without any interruption by an experimenter (Video S1).

## 5. Conclusions

Here, we proposed a soft, miniaturized low-power implantable wireless optoelectronic device with a novel actuating mechanism enabled by a reed switch. A customized control logic circuit including a reed switch enabled a wireless operation with a several ten-folds reduction in power consumption, required for a dual-channel operation. In vitro and in vivo validations of the proposed technology demonstrated the utility and versatility in its function. Such miniaturized implantable dual-channel HF wireless optoelectronic devices, paired with a self-tracking magnetic resonant coupling wireless power transmission system, can stimulate and/or inhibit neuronal activities in a freely-behaving animal. When combined with advanced RF control strategies, the platform can yield potent capabilities in the independent and/or simultaneous control of animals numbering up to five in a cage or multi-cages, which makes it ideal for complex behavior experiments such as the investigation of the social behavior of groups of animals.

## Figures and Tables

**Table 1:** Step by step procedures for the fabrication of the low-power, wireless optoelectronic devices.

Process	Purpose	Equipment/Tools (Model Name/Manufactures)	Progress ^1^
1. Preparation of Cu/PI film	Preparation of the films for defining patterns	PI/Cu film (AC181200R, DupontTM Pyralux^®^) glass Kapton tape	5%
2. UV photo-lithography	Define the patterns	Cleanroom (AggieFab) Spin-coater (G3 P-8, Specialty Coating Systems) Hotplate (SP88850200, Fisher Scientific^TM^) Mask aligner (EVG^®^610, EV Group) Photoresist (AZ 1518, AZ^®^) Developer (AZ Developer 1:1, AZ^®^) Copper etchant (Alfa Aesar™)	30%
3. Components soldering	Mount electrical components on the pattern	Microscope (SPZV 50E, AVEN) Solder cored wire flux (397952, Multicore^®^) Solder stations (WD1002/WP80, Weller) Stainless steel wire (75,000 psi tensile strength, and 0.008’’ diameter, McMaster-Carr) UV epoxy (MED-OG198-54, Epoxy Technology)	60%
4. 1st testing	Function verification	Transmit power system (FEIG electronic) Magnets	70%
5. PDMS encapsulation	Device packaging	Vacuum ovens (Accu Temp 1.9, AI) PDMS (Sylgard^TM^ 184 silicone elastomer kit, Dow^®^)	95%
6. 2nd testing	Device validation	Transmit power system (FEIG electronic) Magnets	100%

^1^**Progress (%)** is based on the step-wise required time per total required time.

## Supplementary Materials

The following are available online at https://www.mdpi.com/1424-8220/20/13/3639/s1, Figure S1: Magnet induced dual-channel device layout and a table of components, Figure S2: Magnet-free, dual-channel device layout and a table of components, Figure S3: Magnet induced dual-channel device signal-flows, Figure S4: Magnet-free, dual-channel device signal-flows, Figure S5: Measurements of durability and heat dissipation, Table S1: Summary of comparison in wireless optoelectronic devices and TX system, Video S1: Magnet induced dual-channel device implant (in vivo), Video S2: Magnet-free, dual-channel device implant (in vivo), Video S3: Mice in open field cage (wireless coverage).
