# Supplementary material for: Fully Implantable Low-Power High Frequency Range Optoelectronic Devices for Dual-Channel Modulation in the Brain
Source: Sensors (Basel). 2020 Jun 29;20(13):3639. doi: 10.3390/s20133639 (PMC7374344; doi:10.3390/s20133639)
Supplement: Supplementary file 1 [file sensors-20-03639-s001.zip › Figure S_final.pdf]

Figure S1

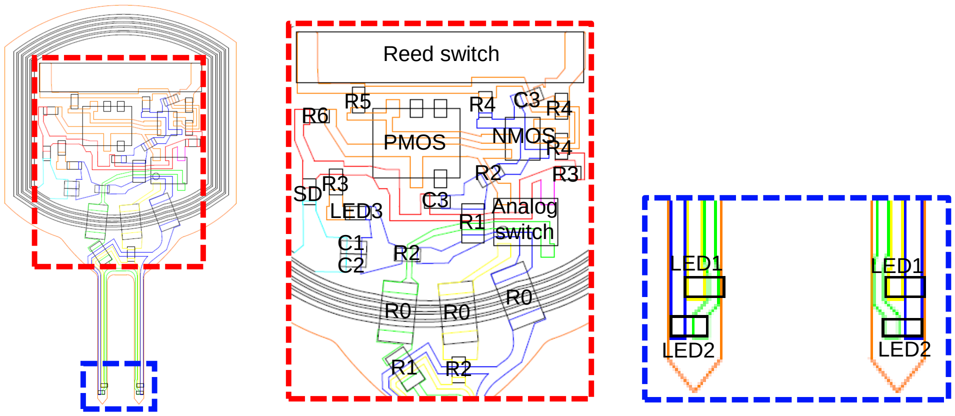

	Components	Product number	Vendor information
**LED1**	527nm, 220 µm x 270 µm x 50 µm	C527TR2227	Cree
**LED2**	460nm, 220 µm x 270 µm x 50 µm	C460TR2227	Cree
**LED3**	632nm, 0.65 mm x 0.35 mm x 0.2 mm	APG0603SEC-E-TT	Kingbright
**SD**	Schottky Diode, 0.65 mm x 0.35 mm x 0.3 mm	DSR01S30SL	Toshiba
**R0**	0 ohm, 1.6 mm x 0.9 mm x 0.55 mm	RCWPM-0603	VISHAY
**R1**	0 ohm, 1.00 mm x 0.55 mm x 0.35 mm	RCWPM-0402	VISHAY
**R2**	0 ohm, 0.65 mm x 0.35 mm x 0.25 mm	RCWPM-0201	VISHAY
**R3**	499 ohm, 0.6 mm x 0.3 mm x 0.23 mm	RC0603F4990CS	Samsung Electro-Mechanics
**R4**	249 kohm, 0.6 mm x 0.3 mm x 0.23 mm	RC0603F2493CS	Samsung Electro-Mechanics
**R5**	10 kohm, 0.6 mm x 0.3 mm x 0.23 mm	RC0603J103CS	Samsung Electro-Mechanics
**R6**	20 kohm, 0.6 mm x 0.3 mm x 0.23 mm	RC0603F203CS	Samsung Electro-Mechanics
**C1**	82 pF, 0.6 mm x 0.3 mm x 0.33 mm	CL03C820JA3NNNC	Samsung Electro-Mechanics
**C2**	330 pF, 0.6 mm x 0.3 mm x 0.33 mm	CL03B331KA3NNNC	Samsung Electro-Mechanics
**C3**	1 µF, 0.6 mm x 0.3 mm x 0.33 mm	CL03A105KP3NSNC	Samsung Electro-Mechanics
**Reed Switch**	7.0 mm x 2.2 mm x 1.6 mm	MK24-A-2	Standex Meder electronics
**NMOS**	1.0 mm x 1.0 mm x 0.34 mm	NTUD3170NZ	ON Semiconductor
**PMOS**	1.7 mm x 1.7 mm x 0.6 mm	NX3008PBKV	Nexperia
**Analog Switch**	1.6 mm x 1.2 mm x 0.5 mm	SN74LVC1G3157	Texas Instruments

Figure S1. Device layout (top) and a table of components used for the low power, magnet induced wireless optoelectronic implant (bottom)

Figure S2

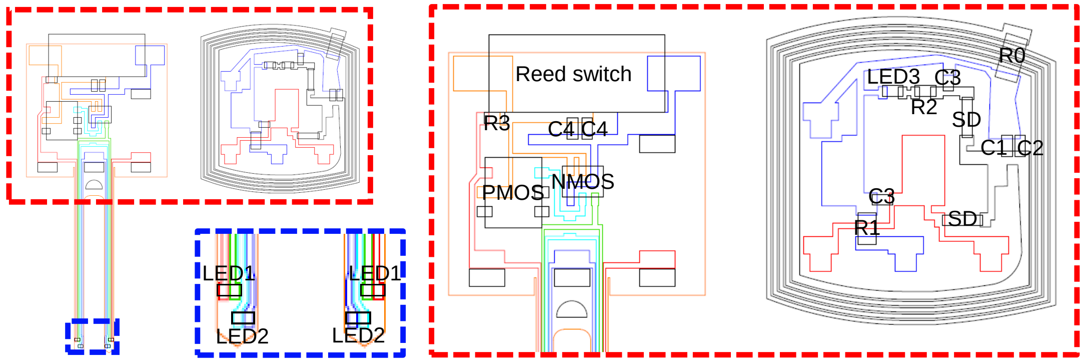

	Components	Product number	Vendor information
**LED1**	527nm, 220 µm x 270 µm x 50 µm	C527TR2227	Cree
**LED2**	460nm, 220 µm x 270 µm x 50 µm	C460TR2227	Cree
**LED3**	632nm, 0.65 mm x 0.35 mm x 0.2 mm	APG0603SEC-E-TT	Kingbright
**SD**	Schottky Diode, 1.4 mm x 0.6 mm x 0.52 mm	DB27309	Panasonic
**R0**	0 ohm, 1.6 mm x 0.9 mm x 0.55 mm	RCWPM-0603	VISHAY
**R1**	0 ohm, 1.00 mm x 0.55 mm x 0.35 mm	RCWPM-0402	VISHAY
**R2**	499 ohm, 0.6 mm x 0.3 mm x 0.23 mm	RC0603F4990CS	Samsung Electro-Mechanics
**R3**	20 kohm, 0.6 mm x 0.3 mm x 0.23 mm	RC0603F203CS	Samsung Electro-Mechanics
**C1**	82 pF, 0.6 mm x 0.3 mm x 0.33 mm	CL03C820JA3NNNC	Samsung Electro-Mechanics
**C2**	330 pF, 0.6 mm x 0.3 mm x 0.33 mm	CL03B331KA3NNNC	Samsung Electro-Mechanics
**C3**	1 µF, 0.6 mm x 0.3 mm x 0.33 mm	CL03A105KP3NSNC	Samsung Electro-Mechanics
**C4**	0.1 µF, 0.6 mm x 0.3 mm x 0.33 mm	CL03A104KP3NNNC	Samsung Electro-Mechanics
**NMOS**	1.0 mm x 1.0 mm x 0.34 mm	NTUD3170NZ	ON Semiconductor
**PMOS**	1.7 mm x 1.7 mm x 0.6 mm	NX3008PBKV	Nexperia
**Reed Switch**	7.0 mm x 2.2 mm x 1.6 mm	MK24-A-2	Standex Meder electronics

Figure S2. Device layout (top) and a table of components used for the low power, RF induced wireless optoelectronic implant (bottom)

Figure S3

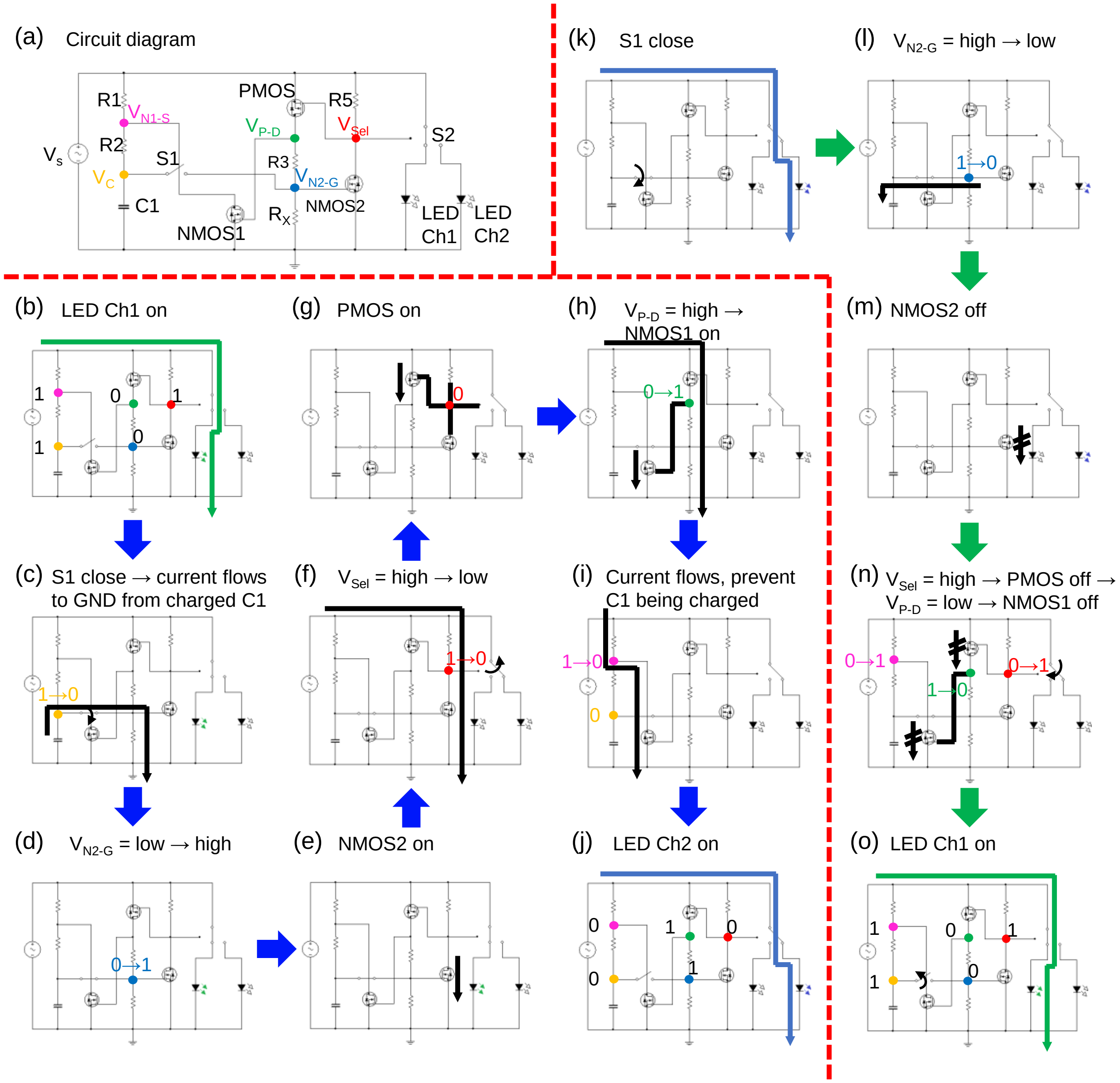

Figure S3. (a) Circuit diagrams of a control logic circuit including a reed switch and LEDs. Reed switch and an analog switch IC are denoted by S1 and S2, respectively. V_{P-D}, $V_{N2-G,}$ and V_{Sel} represent a level of voltages at PMOS drain, NMOS2 gate, and a selection input port of an analog switch, respectively. The rest of parameters for capacitors and resistors are C1 = 1μF, R1 = R2 = 249kΩ, R3 = 10kΩ, R5 = 20kΩ. (b)-(j) Signal-flows LED ch1 to LED ch2. (k)-(o) Signal-flows from LED ch2 to LED ch1.

Figure S4

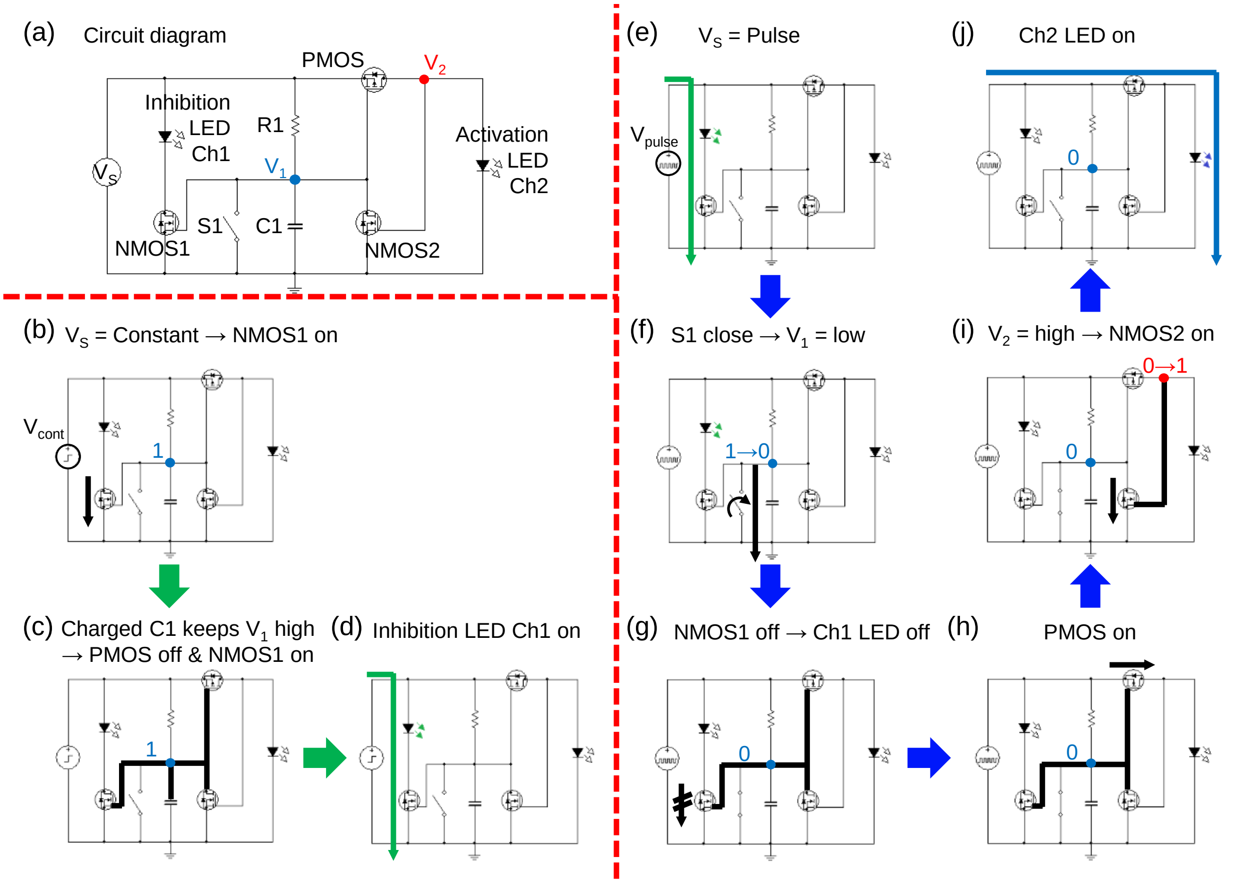

Figure S4. (a) Circuit diagrams of a dual channel device that offers an advanced magnet-free operational mode-inhibition and stimulation of neural activity. Here, a dual channel device automatically activates a channel in response to signals from a remotely located wireless TX system. R1 = 20kΩ and a reed switch is denoted by S1. (b)-(d) Signal-flows from a power supply to Ch1 LED for Ch1 activation. (e)-(j) Signal-flows during switching from Ch1 to Ch2.

Figure S5

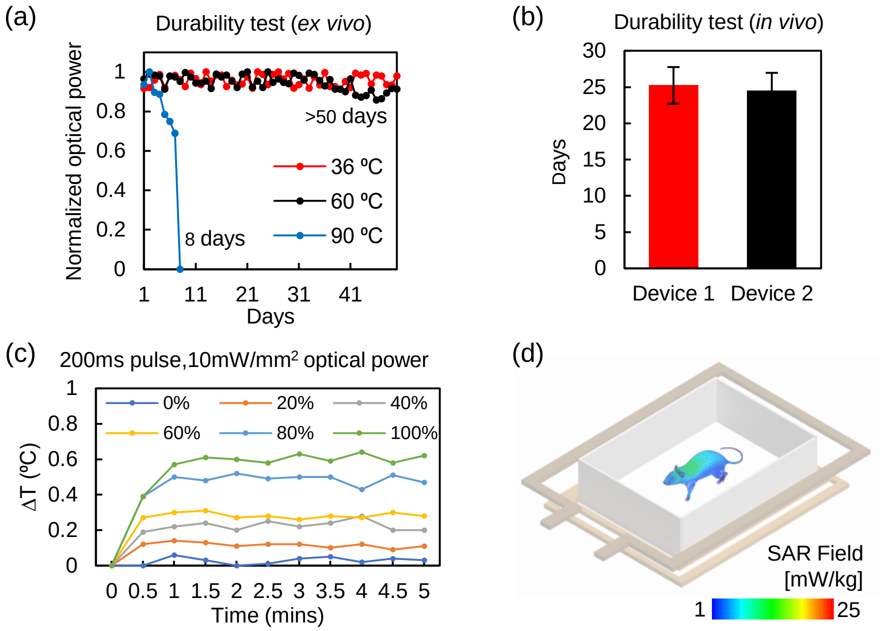

Figure S5. (a) Normalized optical power from devices immersed in 10% PBS at various temperatures *ex vivo*. (b) Implanted device's functional test *in vivo;* low power, magnet induced dual-channel optoelectronic device denoted Device 1 (n=8) and magnet-free, dual-channel optoelectronic device denoted Device 2 (n=8). Bar graphs are mean ±SEM. (c) Measurements of temperature changes in 10% PBS at various duty cycles. (d) The computed SAR distributions on a mouse model with an experimental assay.
