# Supplementary material for: Fully Implantable Low-Power High Frequency Range Optoelectronic Devices for Dual-Channel Modulation in the Brain
Source: Sensors (Basel). 2020 Jun 29;20(13):3639. doi: 10.3390/s20133639 (PMC7374344; doi:10.3390/s20133639)
Supplement: Supplementary file 1 [file sensors-20-03639-s001.zip › Table S_final.pdf]

Table S1

Table S1. Summary of comparison in wireless optoelectronic devices and TX system.

Ref	Device dimension	Full implantation	Target region	Channel number	Cage dimension	Resonance Frequency	TX power level
**6**	10-25 mm^3	O	brain, spinal, peripheral	single	21-cm diameter<br>15-cm height	1.5 GHz	Not mentioned<br>(use power amplifier)
**10**	9.8 mm diameter	O	brain	single	30 × 30 cm	13.56 MHz	12 W
**11**	3.5 × 8.5 × 2.4 mm	X	brain	single	30 × 30 cm	920 MHz	Not mentioned<br>(use power amplifier)
**12**	4.3 × 8 × 0.7 mm	O	brain	multi	16 × 25 × 13 cm	2.3-2.7GHz	Not mentioned<br>(use power amplifier)
**14**	9.8 mm diameter<br>3 mm height	O	Sciatic nerve	multi	10 × 33 × 10 cm	13.56 MHz	12 W
**15**	19 × 14 mm	O	Spinal cord, Sciatic nerve	multi	20 × 20 × 20 cm	13.56 MHz	12 W<br>(use power amplifier)
**16**	3 cm diameter	O	Bladder	multi	30 × 60 × 10 cm	13.56 MHz	Not mentioned
**35**	10 × 10 mm	O	brain	multi	16 × 25 × 13 cm	13.56 MHz	4 W
**Ours**	9 × 8.5 mm	O	brain	multi	16 × 25 × 13 cm<br>28 × 28 × 30 cm	13.56 MHz	2 W
